# Characterization of GmMATE13 in its contribution of citrate efflux and aluminum resistance in soybeans

**DOI:** 10.3389/fpls.2022.1027560

**Published:** 2022-10-21

**Authors:** Zhengbiao Wang, Yuanqi Liu, Wenmo Cui, Li Gong, Ying He, Qingxiu Zhang, Xiangxiang Meng, Zhenming Yang, Jiangfeng You

**Affiliations:** Jilin Province Engineering Laboratory of Plant Genetic Improvement, College of Plant Science, Jilin University, Changchun, China

**Keywords:** aluminum resistance, resistance mechanism, multi-drug and toxic compound extrusion family, citrate transporter, Glycine max

## Abstract

Citrate exudation mediated by a citrate transporter of the MATE protein family is critical for resisting aluminum (Al) toxicity in soybeans. However, the expression patterns of citrate transporter genes differ under Al stress. Thus, exploring the responsive pattern of GmMATEs in response to Al stress is of great importance to understand the Al resistance mechanism in soybeans. In the present study, the phylogenetic analysis, transcriptionally expressed pattern, and function of *GmMATE13* were investigated. The results show that soybean GmMATE13 is highly homologous to known citrate transporter proteins from other plants. Under Al exposure, the transcript abundance of *GmMATE13* was increased during a 24 h Al treatment period. The expression of *GmMATE13* is specifically induced by Al exposure, but not by the status of Fe, Cu, Cd, or La. Moreover, it was also highly increased when soybean seedlings were grown on acidic soil with a high Al content. Subcellular localization showed that GmMATE13 was localized on the plasma membrane when it was transiently expressed in *Arabidopsis* protoplasts. Investigation of tissue localization of GmMATE13 expression by investigating GUS activity staining under control of the GmMATE13 promoter showed that it was mainly expressed in the central cylinder in the root tips of the soybean under Al-free conditions, yet extended to cortical and epidermis cells under Al stress. Finally, overexpressing *GmMATE13* in soybean hairy roots enhanced Al resistance by increasing citrate efflux. Collectively, we conclude that *GmMATE13* is a promising candidate to improve the resistance of soybean to Al toxicity in acidic soil.

## Introduction

Unlike manganese, zinc, iron (Fe), copper, and molybdenum, aluminum (Al) is not essential for most creatures though it is the most abundant metal element in the earth’s crust (Rengel, 2004). Aluminum exists in toxic soluble ionic forms in acidic soil and inhibits the growth of plant roots, which decreases the absorption capability of plant roots for nutrients and water, ultimately leading to crop yield reduction (Kochian et al., 2005). Micromolar concentration of Al^3+^ can inhibit root elongation of many crop species within minutes of exposure (Kochian, 1995). The root tip is the most sensitive target of Al toxicity in plants, especially the transition zone located between the elongation zone of roots and the apical meristem (Sivaguru and Horst, 1998). Al can interact with the cell wall, plasma membrane, and symbionts of root cells (Ma, 2000). Meanwhile, Al has a high binding affinity with oxygen-supplying compounds, such as phospholipids, nucleotides, carboxylic acids, proteins, DNA, RNA, and inorganic phosphates (Martin et al., 1988), which can damage the structure and function of root cells and inhibit root elongation.

Some plants have evolved multiple mechanisms to tolerate Al stress, helping plants grow normally in acidic soils. Of them, Al-induced organic acid secretion is one of the most important Al-resistance mechanisms (von Uexküll and Mutert, 1995; Yang et al., 2000; Kochian et al., 2004). The species of Al-induced secretion of organic acid anions include citrate, oxalate, and malate, which form strong complexes with Al^3+^ to protect root cells. For instance, wheat (*Triticum aestivum*) can resist Al by secreting malate under Al stress (Delhaize et al., 1993). Maize (*Zea Mays*) (Pellet et al., 1995), *Cassia Tora* (Ma et al., 1997a), and soybeans (*Glycine Max*) (Yang et al., 2001) are resistant to Al by secreting citrate. Buckwheat (*Fagopyrum esculentum Moench*) (Ma et al., 1997b) and taro (*Colocasia esculenta*) (Ma and Miyasaka, 1998) resist Al by secreting oxalate. In some cases, there are two different organic acid anions secreted by plant roots to respond Al toxicity. For example, oilseed rape (*Brassica Napus*), maize, *Avena sativa*, *Raphanus Sativus*, and *Secale cereale*, simultaneously release malate and citrate, whereas the roots of *Amaranthus hypochondriacus* secrete oxalate and citrate under Al stress (Pellet et al., 1995; Fan et al., 2016).

The multidrug and toxic compound extrusion (MATE) family is a secondary transporter family, mainly involved in transporting secondary metabolites, such as alkaloids (Shoji et al., 2009), flavonoids (Debeaujon et al., 2001), and anthocyanins (Pérez-Díaz et al., 2014). The members of this family are demonstrated to be implicated in detoxification of toxic compounds and heavy metals (Diener et al., 2001), modulation of disease resistance (Sun et al., 2011), outflow of plant hormones (abscisic acid) (Zhang et al., 2014), Fe transport (Durrett et al., 2007), and toleration of Al toxicity (Furukawa et al., 2007). The matrix transport is mediated by MATE transporters coupled with the electrochemical gradient of transmembrane cations, such as H^+^ or Na^+^ ions (Kuroda and Tsuchiya, 2009; Shoji, 2014). The MATE transporter was revealed to have a unique topology of 12 transmembrane (TM) helices, which is different from any other known transporters (He et al., 2010).

Furukawa et al. (2007) identified a MATE family gene *HvAACT1* that encodes a plasma membrane-localized citrate transporter, which is responsible for Al activated-citrate secretion from the barley root apex. Magalhaes et al. (2007) identified the aluminum-resistant citrate secretion gene *SbMATE* from sorghum by the method of map-based cloning, which can improve the secretion of citrate in sorghum and the Al resistance of sorghum. Later, AtMATE (Liu et al., 2009); ZmMATE1 and ZmMATE2 (Maron et al., 2010); TaMATE1-4A, TaMATE1-4B, and TaMATE1-4D (Tovkach et al., 2013); VuMATE1 (Yang et al., 2011); VuMATE2 (Liu et al., 2018); FeMATE1 and FeMATE2 (Lei et al., 2017); and GmMATE75, GmMATE79, and GmMATE87 (Zhou et al., 2019) were successively characterized as citrate transporters to confer Al-activated citrate efflux. Additionally, the spatial expression of citrate transporters determined their roles in Al detoxification or Fe translocation. The initial role of HvAACT1 is to transport citrate to the xylem of the root and be involved in Fe translocation to the mature zone of the root by forming ferric citrate complexes (Fujii et al., 2012). Transposon of 1 kb insertion upstream of 5’UTR promotes the secretion of citrate from the root to the rhizosphere to detoxify Al (Fujii et al., 2012). The transposon-like element in cv Carazinho displaying citrate secretion extends the expression of *TaMATE1B* to the root tip, where it provides citrate efflux and enhances Al resistance (Tovkach et al., 2013). The temporal and spatial expression patterns of MATE genes also determine its distinct biological functions during process of citrate secretion and then Al resistance (Lei et al., 2017; Zhou et al., 2019). For instance, in rice bean, buckwheat, sorghum, barley, and other plant species, different MATE confers different roles in citrate secretion patterns (Magalhaes et al., 2007; Yang et al., 2011; Lei et al., 2017). Al stress can differentially regulate two citrate transport systems. The secretion of citrate from the root tip of rice bean induced by Al was biphasic, including a small amount of citrate in the early stage mediated by VuMATE2 and a large amount in the late stage mediated by VuMATE1. Without Al stress, VuMATE1 is not transcribed, and at least 3 h of Al exposure is required to begin the induction of its expression (Liu et al., 2013). However, VuMATE2 showed a structural expression pattern with a sustained increase in expression levels after 0.5 to 2 h of Al exposure and relatively stable expression thereafter (Liu et al., 2018). Al-induced expression of VuMATE1 and VuMATE2 occurs through diverse signal transduction pathways (Liu et al., 2018). In buckwheat, FeMATE1 and FeMATE2 play different roles in response to Al. FeMATE1 may be involved in Al-induced citrate efflux from buckwheat roots, whereas FeMATE2 may be responsible for the Golgi-associated internal detoxification of Al (Lei et al., 2017).

From the soybean genome, there were 117 genes encoding MATE transporters identified. Based on sequence similarity with another 14 MATE transporters from other plant species, eight soybean MATE proteins in subgroup C4-3 were predicted as citrate transporters to be involved in Al detoxification or Fe translocation in soybeans (Liu et al., 2016). GmMATE75, GmMATE79, and GmMATE87 were identified as the plasma-membrane-localized citrate transporters, and overexpression of them in soybean hairy roots and *Arabidopsis* driven by 35S promoter can increase citrate efflux, decrease Al accumulation, and alleviate root elongation inhibition. However, the three *GmMATEs* are quite different in expression pattern and tissue-localization, therefore, playing different roles in Al-induced citrate efflux and protection of the roots from Al toxicity (Zhou et al., 2019). Al treatment extended the expression of *GmMATE75* and *GmMATE79* from the central cylinder to cortical and epidermis cells in soybean transgenic hairy roots, but the expression of *GmMATE87* was restricted to the central cylinder irrespective of Al treatment. Obviously, more GmMATEs deserved to be characterized to better understand the strategies of soybeans to deal with Al stress. In this present study, GmMATE13 was characterized in terms of its role in Al resistance, emphasizing the link between expression pattern and biological function.

## Material and methods

### Plant materials and growth conditions

Seeds of Al-resistant soybean variety Jiyu 70 were sterilized with 1.0% sodium hypochlorite and planted in a mixture of soil and vermiculite at a ratio of 3:1. The seeds were cultured under 25°C dark for about 3 days until germination, transplanting, and incubating the seedlings for 1 week in nutrient solution (pH 4.5) as described previously (Horst et al., 1992). Continuous aeration was required, and nutrient solution was replaced every 2 days. After 7 days cultivation, roots were washed overnight with 0.5 mM CaCl_2_ solution at pH 4.5.

For the time course experiment, 0.5 mM CaCl_2_ solution plus 30 µM AlCl_3_ (pH 4.5) were treated for 0, 4, 6, 12, and 24 h, respectively, and root tip 0-1 cm was taken. For the other metal stresses, 0.5 mM CaCl_2_ with pH 4.5 (including 10 µM La^3+^, 25 µM Cd^2+^, 30 µMAl^3+^, or 1 µM Cu^2+^) for 4 h were used, excising root tip 0-1 cm, respectively. In the Fe deficiency assay, soybean seedlings were cultured in nutrient solution (Horst et al., 1992) without Fe (pH 4.5) for 5 days, and 0-1 cm of root tip was excised by a scalpel.

### The sequence analysis and gene cloning of *GmMATE13*

Sequences of GmMATE13 (Glyma.02G181800.1) were searched from NCBI (https://www.ncbi.nlm.nih.gov). Software MEGA5 was used to generate a phylogenetic tree of GmMATE13 and other identified citrate transporters. The cDNA template was prepared with the root apices of Jiyu 70 after 4 h Al treatment. The sequences of GmMATE13 were cloned by reverse transcription PCR. The sequence was aligned in DNASTAR.

### Quantitative real-time PCR (qRT-PCR)

The reaction system and protocol of qRT-PCR were described in previous literature (Zhou et al., 2019). Gene-specific primers are shown on **Table S1**. The internal standard is *GmTubulin* (GenBank ID: 100811275). The relative *GmMATE13* transcriptional expression level was calculated by the 2^-ΔΔCt^ method as described (Livak and Schmittgen, 2001).

### GmMATE13 subcellular localization

The CDS of *GmMATE13* was constructed into the pENSG-N-GFP vector with a CaMV 35S promoter. The GFP control and GFP-GmMATE13 fusion proteins were transiently transformed into *Arabidopsis* protoplasts. The transformed protoplast cells were stained by marker (Cell Mask™ Orange plasma membrane stain, C10045, USA). GFP fluorescence was observed by confocal microscopy (Zeiss, LSM 900 with Airyscan 2, Germany).

### Overexpressing *GmMATE13* in soybean hairy roots

The CDS of *GmMATE13* was constructed in the pCAMBIA3301 vector with CaMV 35S as a promoter and nanoluciferase as the label and then diverted into the Agrobacterium strain K599. The Jiyu 62 cotyledons were transformed with strain K599 according to Subramanian et al. (2005). The luciferase activity in soybean was confirmed *via* luminometer (Centro LB960XS3, Bert-hold, Germany) with substrate Coelenterazine (Prolume Ltd., Pinctop, USA) to verify the success rate of transformants. The luciferase value ≥5000 of hairy roots was identified as a successful transformation (*GmMATE13-OE*) and then selected for next experiment. Hairy roots transformed by only K599 were the wild-type (WT) control. Then, the WT and *GmMATE13-OE* hairy roots were cultured in the 0.5 mM CaCl_2_ solution plus 0 or 30 µM AlCl_3_ (pH 4.5) in a container. After 4 h, collecting the root exudates to measure citrate efflux *via* the enzymatic method according to Delhaize et al. (1993). Meanwhile, excising 0-1 cm root apices to measure Al content, which were extracted by 2 M HCl and detected *via* inductively coupled plasma mass spectrometry (ICP-MS) (Agilent Technologies 7500C, USA). Callose of soybean hairy roots (0-3 cm) was extracted and then evaluated according to a previous study (Wang et al., 2021). Briefly, the roots were fixed in ethanol and homogenized in NaOH (1 M), incubated in a water bath 80°C for 30 min and then centrifuged. The supernatant (200 µL) was mixed with 0.1% aniline blue solution (400 µL), HCl (1 M, 210 μL), glycine buffer (1 M, pH 9.5, 590 μL) in a water bath at 50°C for 20 min. The callose content was determined using fluorescence spectrophotometry (excitation wavelength 400 nm; emission wavelength 500 nm) with laminarin as a standard.

### Tissue-localization of the expression of *GmMATE13*

The promoter sequence of *GmMATE13* was cloned from Jiyu70, Jiyu62, Williams82 genomic DNA. The cloned sequences (1500 bp) did not differ at all and then were constructed into pCAMBIA3301 vector with GUS as the label and diverted into the Agrobacterium strain K599. The cotyledon transformation is the same as the previous step. With exposure to 0.5 mM CaCl_2_ solution plus 0 or 30 µM AlCl_3_ at pH 4.5, the same growing roots were dipped in a mixture X-gluc solution to stain and photographed with microscope. Using the microtome to dissect slices of the root apex to 10 μm, they put them on the glass slides, which were observed *via* microscope (Zeiss 2012 Observer A1, Göttingen, Germany).

### Transcriptional expression analysis of *GmMATE13*

For analysis of the tissue location of expression of *GmMATE13* in different types of soil, Jiyu 70 were grown on different types of soil with varied pHs as weak acidic (Changchun, Jilin), acidic (Wenzhou, Zhejiang), weak alkaline (Liangcheng, Inner Mongolia), and strong acidic (Changsha, Hunan). The information of pH, exchange Al and Fe are listed within **Table S1**. After 15 days cultivation, root apices of 0-3 cm, stems, and leaves were sampled and stored in a centrifuge tube, respectively, to analyze the transcriptional expression of *GmMATE13.*

### Statistical analysis

All of the data were analyzed by IBM SPSS, and *P*<.05 represented statistically significant.

## Results

### Phylogenetic analysis of GmMATE13

**Figure 1:**
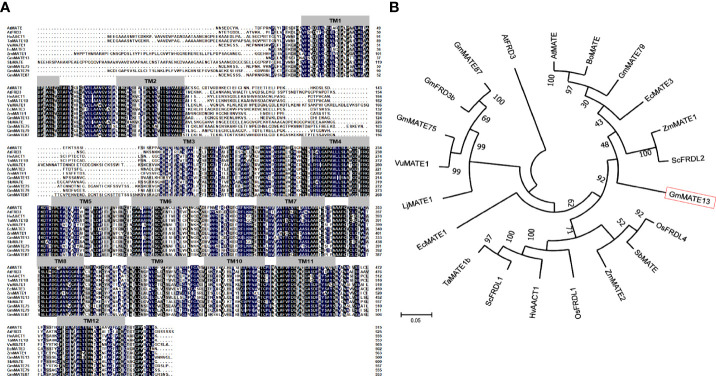
Comparison and phylogenetic relationship of GmMATE13 with citrate transporter proteins of other species. **(A)** Multiple sequence amino acid alignment of GmMATE13 and other known citrate transporter proteins. Strictly conserved amino acids are highlighted with black. **(B)** Phylogenetic tree analysis of GmMATE13 and other known citrate transporter proteins.

The GmMATE13 amino acid sequence was highly conserved with other citrate transporters and contained 12 transmembrane domains named TM1-TM12. There was a loop of 73 amino acids between TM2 and TM3. There was a large variation in the N-terminal among these citrate transporters (**Figure 1A**).

MEGA5.0 software was used for phylogenetic analysis of MATE proteins from different species, and the results are shown in **Figure 1B**. GmMATE13 has the highest sequence homology with EcMATE3, which is shown to function as a citrate transporter involved in Al resistance in plants (Sawaki et al., 2013). ZmMATE1, ZmMATE2 (Maron et al., 2010; Maron et al., 2013), GmMATE87 (Zhou et al., 2019), AtMATE (Liu et al., 2009; Liu et al., 2012), BoMATE (Wu et al., 2014), and ScFRDL2 (Yokosho et al., 2010) also had high homology with GmMATE13, and these proteins are proved to be involved in Al resistance in plants. The homology of GmMATE13 with ZmMATE1, ZmMATE2, AtMATE, BoMATE, and ScFRDL2 was 52.43%, 53.17%, 60.19%, 59.20%, and 56.32%, respectively. The homology of the GmMATE13 protein sequence with GmMATE79, GmMATE87, GmMATE47, and GmMATE75 was 50.36%, 56.19%, 51.75%, and 49.64%, respectively.

### Transcriptional expression pattern of *GmMATE13*

**Figure 2:**
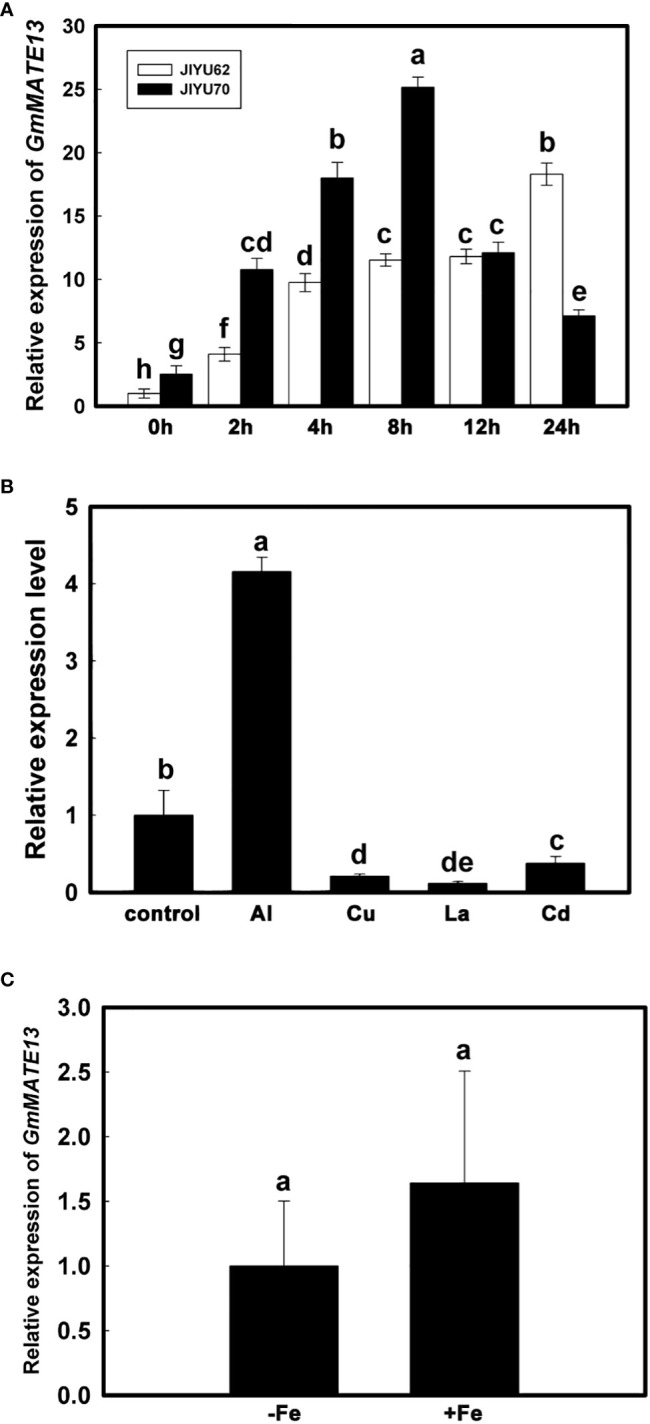
Transcriptional expression pattern analysis of *GmMATE13*. **(A)** Time course of *GmMATE13* expression patterns in root tips under aluminum stress. After 7 days, the seedlings (Jiyu70 and Jiyu62) were transplanted to 0.5 mM CaCl_2_ solution plus 30 µM AlCl_3_ at pH 4.5. The 0-1 cm root tips were excised after 0, 2, 4, 8, 12, and 24 h Al exposure to investigate the expression of *GmMATE13*. **(B)** Effect of different metals on the expression of *GmMATE13* in root apices. Seedlings were treated to 30 µM Al, 1 µM Cu, 10 µM La, or 25 µM Cd for 4 h, excising the 0-1 cm root tips. **(C)** Transcriptional expression of *GmMATE13* with -Fe solution. The seedlings were cultivated in nutrient solution with or without Fe-EDTA for 10-day culture, excising the 0-1 cm root apices. Data are represented as means ± SD (*n*=3). Error bars with different letters represented significantly different by Tukey’s test (*P*<.05).

*GmMATE13* was expressed in the root tips of both Al-resistant soybean variety Jiyu 70 and Al-sensitive soybean variety Jiyu 62, but the relative expression level was different between the two varieties. The expression level of Jiyu 70 was higher than Jiyu 62 along with Al. With the extension of treatment time, the expression level of *GmMATE13* increased gradually in the root tips of Jiyu 62, remained stable from 4 to 12 h, and suddenly increased at 24 h. By contrast, in the root tips of Jiyu 70, with the extension of Al treatment time, the expression level of *GmMATE13* increased first and then decreased and reached the maximum at 8 h (**Figure 2A**).

To determine whether the induction of *GmMATE13* expression was Al-specific, we tested the effect of other metals on *GmMATE13* expression in the root apices. As shown in **Figure 2B**, *GmMATE13* expression was induced by Al^3+^ but not other metals (Cd^2+^, La^3+^, and Cu^2+^). Moreover, after 10 days culturing, there was no significant difference in the expression level of *GmMATE13* between the +Fe treatment and the -Fe treatment (**Figure 2C**), indicating that the expression level of *GmMATE13* did not respond to the Fe deficiency.

### Subcellular localization of GmMATE13

To examine the subcellular localization of GmMATE13, we introduced plasmid-containing GFP or GFP-GmMATE13 fusion protein into the Arabidopsis protoplast, and the GFP fluorescence was observed to study the location of GmMATE13. While the GFP control was expressed in both the cytoplasm and plasma membrane, GFP-GmMATE13 was expressed on the plasma membrane, which was overlapped with the plasma membrane marker staining, indicating that GmMATE13 was a plasma membrane-localized protein. The location of GmMATE13 was the same as other identified citrate transporters, such as GmMATE75 (Zhou et al., 2019).

### Overexpressing *GmMATE13* in soybean hairy roots

**Figure 3:**
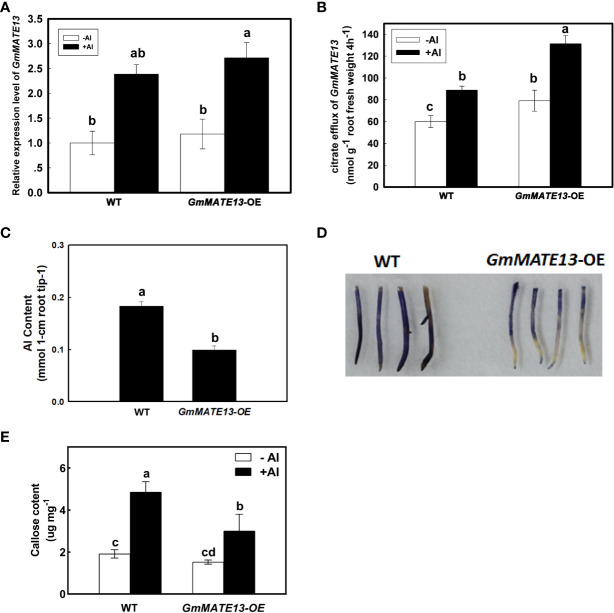
Characteristics of Al resistance in soybean hairy roots overexpressing *GmMATE13*. **(A)** Transcriptional expression of *GmMATE13* in soybean hairy roots of *GmMATE13*-OE. **(B)** Citrate efflux from *GmMATE13*-OE soybean hairy roots. **(C)** Al accumulation in the root apices of *GmMATE13*-OE hairy roots. **(D)** The hematoxylin staining in WT and *GmMATE13*-OE soybean hairy roots under Al stress. **(E)** Callose content in the hair roots of WT and *GmMATE13*-OE without or with 30 µM AlCl3 treatment for 4 **(h)** Values represent the means ± SD (*n*=3). Different letters indicate statistically significant difference by Tukey’ s test (*P*<.05).

To investigate the biological function of GmMATE13, *GmMATE13* was expressed in soybean hairy roots under the control of the CaMV35S promoter. *GmMATE13*-OE hairy roots increased the transcript levels of *GmMATE13* and citrate efflux under either -Al or +Al treatment (**Figures 3A, B**). The Al content of hairy root tips of *GmMATE13-OE* treated with Al was also lower than that of WT (**Figure 3C**). *GmMATE13*-OE roots showed lighter hematoxylin staining after 4 h of Al treatment compared with WT roots, which was consistent with higher citrate efflux and lower Al content in root tips (**Figure 3D**). Overexpression of *GmMATE13* decreased callose concentration in soybean root apices compared with WT (**Figure 3E**). Al treatment increased the callose concentration in WT and *GmMATE13*-OE; however, the callose content of *GmMATE13*-OE remained significantly lower than that of WT, which is consistent with the phenotype of Al resistance in *GmMATE13*-OE soybean hairy roots.

### β-Glucuronidase (*GUS*) staining

**Figure 4:**
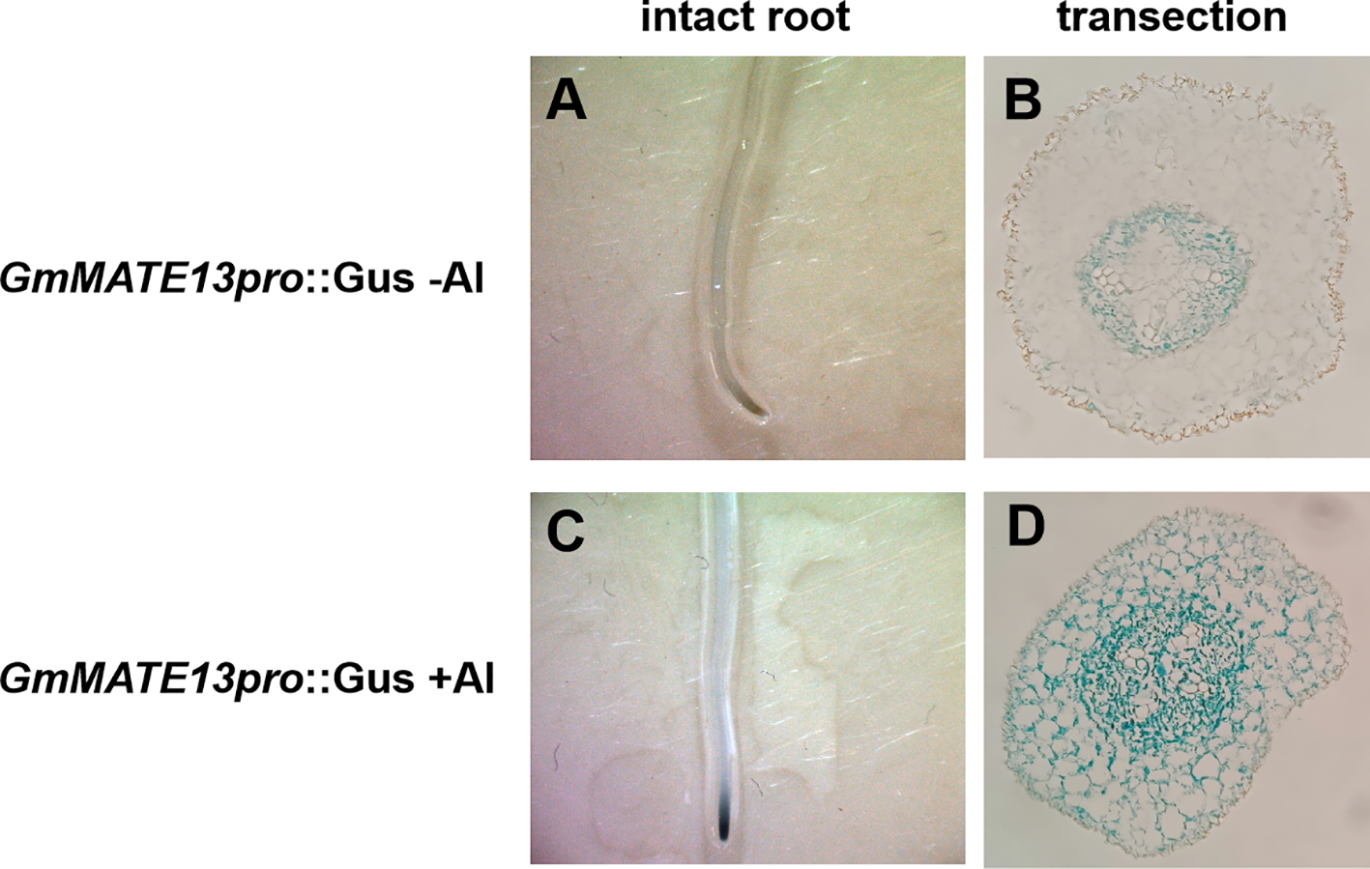
Tissue-level localization of *GmMATE13* expression. Gus staining observed the activation of *GmMATE13* promoter in intact root or its cross-section slices. The promoter of *GmMATE13* without Al stress is shown in **(A, B)** The promoter of *GmMATE13* with Al stress was shown in **(C, D)**.

To study the tissue-localization of the expression of *GmMATE13*, the promoter region of *GmMATE13* fused with the *GUS* gene was introduced into soybean hairy roots. In the absence of Al treatment, GUS staining of *pGmMATE13::GUS* transformation was confined to the central cylindrical region of the root apex (**Figures 4A, B**). Its GUS staining got heavier and extended from the central cylinder to cortical and epidermis cells after 4 h Al treatment (**Figures 4C, D**), which was similar to other *pGmMATEs::GUS* (Zhou et al., 2019). Further observation showed that the expression location of *GmMATE13* was different from the other three genes; namely, it was only expressed in the root tip of soybean hairy roots but had almost no expression in other parts of the hairy roots, indicating that *GmMATE13* may have specificity and high efficiency for soybean Al resistance.

**Figure 5:**
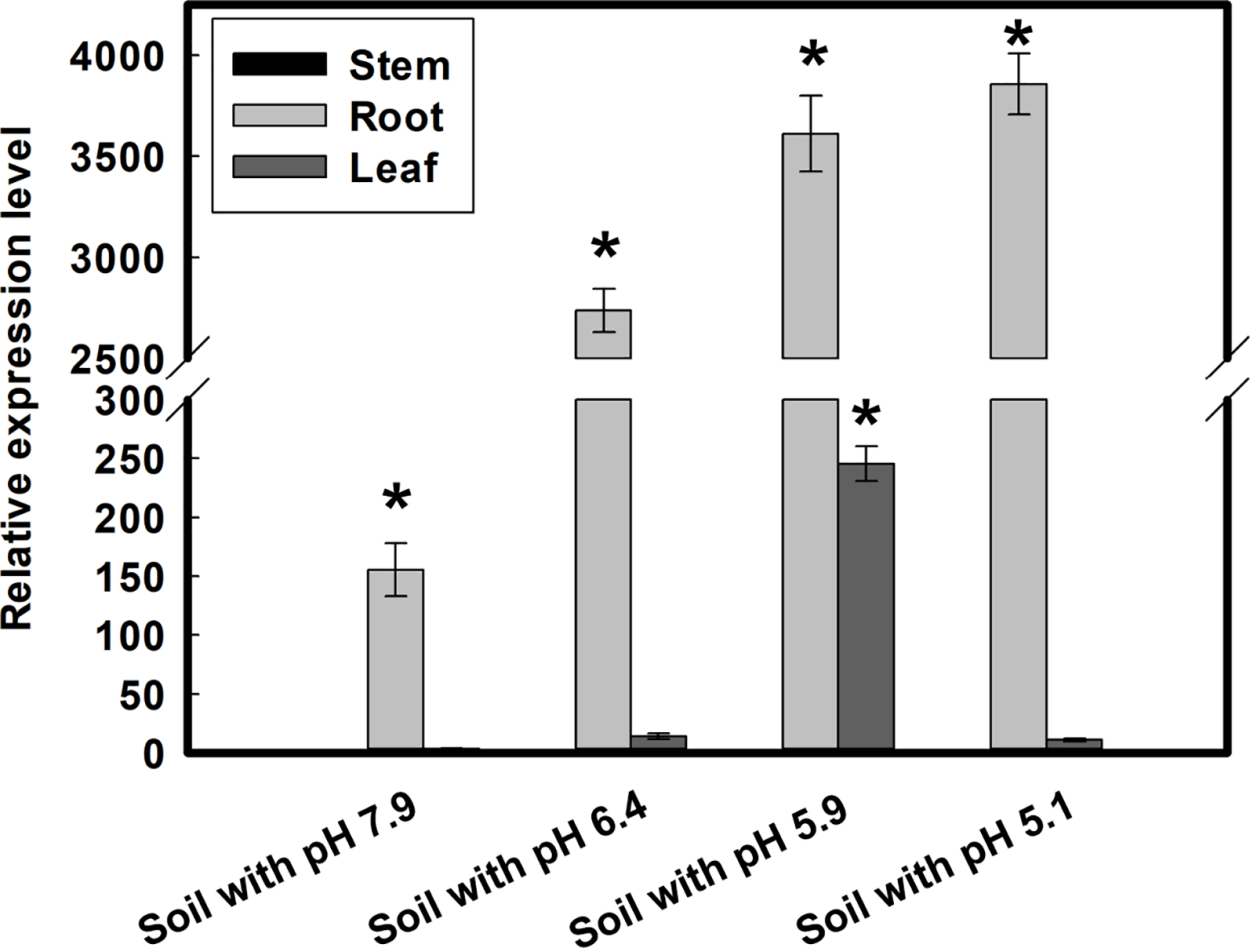
Expression differences of *GmMATE13* in four types of soil. Seedlings were grown in four types of soil for 10 days, and root, stems, and leaves were harvested for measuring the expression of *GmMATE13.* Data are means ± SD (*n*=3). The asterisk (*) represents statistically significant by Dunnett’s *t* test (*P*<.05).

### Expression of *GmMATE13* in soybean grown on soils with different pH

In order to analyze the expression patterns of *GmMATE13*, the soybeans were grown on different soils characteristic of varied pH, exchangeable Al and Fe (**Table S2**). As shown in **Figure 5**, *GmMATE13* is mainly expressed in roots and only weakly in stems and leaves. The expression level of *GmMATE13* is the lowest in the soybeans grown on soils with high pH, low Al, and low Fe conditions and highest in roots on soils of low pH, high Al, and low Fe soils. In addition to Fe and Al, the expression of *GmMATE13* might also be affected by other soil factors.

## Discussion

**Figure 6:**
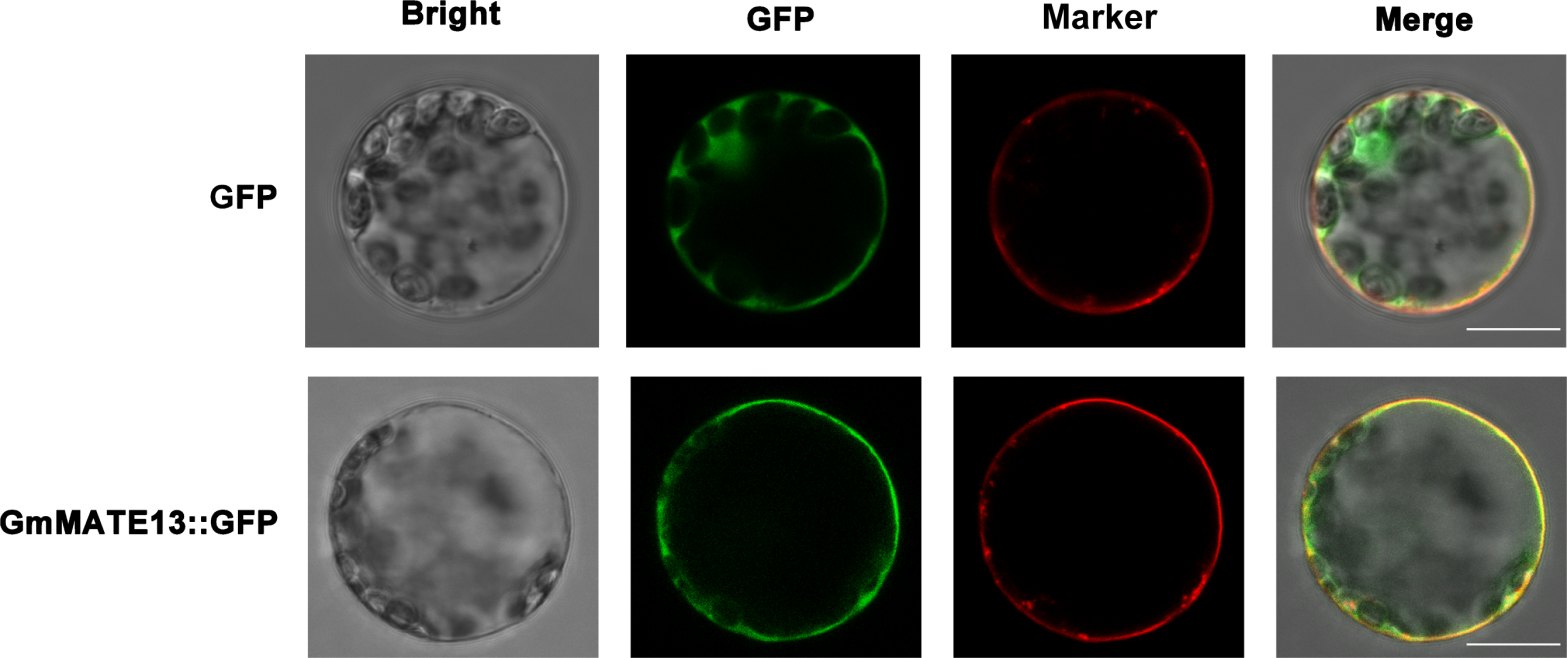
Subcellular localization of GmMATE13. GFP-GmMATE13 fusion proteins and GFP control were transiently transformed in *Arabidopsis* protoplasts. GFP control and GFP-GmMATE13 fusion proteins were shown in the first or two-row images, respectively. The scale bar indicates 10 µm.

Al-induced citrate efflux is one of the most important Al-resistance mechanisms in soybeans (Yang et al., 2000; Yang et al., 2001; Silva et al., 2001). Bioinformatics analysis shows that GmMATE13 proteins belong to the MATE family and are citrate transporters with 12 transmembrane domains and a large loop structure between the second and third domains but with high specificity in the N-terminal sequence (**Figure 1A**). GmMATE13 has high homology with AtMATE, VuMATE, and LjMATE (**Figure 1B**), all located on the plasma membrane (**Figure 6**), which are transporters associated with both Al and Fe (Liu et al., 2012; Liu et al., 2013; Takanashi et al., 2013).

Analysis of the temporal expression pattern of *GmMATE13* showed that *GmMATE13* was induced by Al, and the expression level distinguished between Al-tolerant soybean variety Jiyu 70 and Al-sensitive soybean variety Jiyu 62. The expression of *GmMATE13* showed a trend of first increase and then decrease in Jiyu 70, whereas in Jiyu 62, increasing first, after stabilizing for a period of time, and then increasing again (**Figure 2A**). For a specific experiment, the expression of *GmMATE13* was downregulated by La^3+^, Cu^2+^, and Cd^2+^ (**Figure 2B**), which was a slight difference from other *GmMATEs* (Zhou et al., 2019). The expression level of *GmMATE13* was not induced by Fe deficiency (**Figure 2C**), indicating that *GmMATE13* might not be involved in the Fe transport pathway in soybeans. Previous studies have found that the function of the citrate transporter is not only involved in the Al resistance of plants, but also in the Fe translocation (Liu et al., 2012; Liu et al., 2013; Takanashi et al., 2013; Liu et al., 2016). *HvAACT1* expression is mainly involved in Fe transport in the root and the Al-resistance pathway when the expression of *HvAACT1* migrates to the epidermis (Fujii et al., 2012).

It has been proved that H_2_S acts on the downstream of NO and mediates Al‐induced citrate efflux, attaching resistance to Al toxicity in plants (Wang et al., 2019). The effect of other soil factors on *GmMATE13* gene expression needs to be studied in the future. In the four types of soil, *GmMATE13* was mainly expressed in roots, not in stems, and only weakly in leaves, and the highest expression level was found in the soil with low Fe, high Al, and low pH, indicating that *GmMATE13* showed a response to high Al in highly acid soil and played a key role in detoxifying Al from soybeans (**Figure 5**).

Overexpression of *GmMATE13* under the control of the CaMV 35S promoter in soybean hair roots increased citrate secretion irrespective of being treated with Al or not, and reduced Al content in root tips when exposed to Al (**Figure 3**). Al-induced callose deposition is a sensitive indicator of Al toxicity and has been used as a convenient and rapid screening parameter for Al injury in addition to root elongation measurement (Horst et al., 1997; Yang et al., 2000; Zhang et al., 2015; Wang et al., 2021). Overexpression of *GmMATE13* decreased callose concentration in soybean root apices compared with WT (**Figure 3**). Overexpression of *SbMATE* and *ZmMATE1* in *Arabidopsis thaliana* (Magalhaes et al., 2007; Maron et al., 2010) and *HvAACT1* overexpression in tobacco (Furukawa et al., 2007) showed significantly enhanced citrate secretion and Al resistance. The function of *GmMATE13* was shown to increase Al resistance by heterogeneous expression in Arabidopsis by high Al concentration treatment (400 µM) (Wang et al., 2019). Multiple genes, such as *AtMATE1*, *AtALMT1*, *AtALS3*, and *AtSTOP1*, are involved in the Al-resistance responses in *Arabidopsis* (Hoekenga et al., 2006; Liu et al., 2009; Liu et al., 2014; Huang, 2021). The functional and structural characteristics of GmMATE13 are consistent and resemble those reported for other citrate-permeable MATEs in barley (Furukawa et al., 2007), sorghum (Magalhaes et al., 2007), maize (Maron et al., 2010), and Arabidopsis (Liu et al., 2009).

The physiological functions of plant MATEs reported so far include xenobiotic secretion, accumulation of secondary metabolites such as alkaloids and flavonoids, Fe translocation, Al detoxification, and plant hormone signal transduction, suggesting that MATE transporters are involved in a series of biological events during plant development (Diener et al., 2001; Durrett et al., 2007; Furukawa et al., 2007; Magalhaes et al., 2007; Thompson et al., 2010; Takanashi et al., 2014). Through the tissue-level localization analysis, Zhou et al. (2019) revealed that the expression of *GmMATEs* extended from the central cylinder to cortical and epidermis cells after 4 h Al exposure, and so did *GmMATE13* (**Figures 6B, D**). Interestingly, it only expressed in the root tip of soybean (**Figures 6A, C**), indicating that *GmMATE13* may have specificity and high efficiency for soybean Al resistance.

In conclusion, GmMATE13 was identified in soybean as a plasma-membrane localized citrate transporter. The transcriptional expression of *GmMATE13* was induced by Al stress, and different expression patterns were observed between Al-sensitive Jiyu 62 and Al-resistant Jiyu 70. The overexpression of *GmMATE13* in hairy roots increased the Al resistance of soybeans. There was a high expression of *GmMATE13* in root tips of soybeans grown on acidic soil, indicating that *GmMATE13* play a key role in the soybean Al resistance signaling pathway. The regulation mechanism deserved further investigation.

## Data availability statement

The original contributions presented in the study are included in the article/**Supplementary Material**. Further inquiries can be directed to the corresponding author.

## Author contributions

JY designed the entire experiment. ZW, YL, WC, LG, YH, and QZ performed the major experiments. XM and ZY helped in data analysis and some useful advice in experiment design. JY and ZW wrote and revised the manuscript. All authors read, reviewed, and approved the manuscript.

## Funding

Financial support for this research was provided by the National Natural Science Foundation of China (No. 32172660) for JY, University Undergraduates Innovating Experimentation Project (No. 202210183330) for YL.

## Conflict of interest

The authors declare that the research was conducted in the absence of any commercial or financial relationships that could be construed as a potential conflict of interest.

## Publisher’s note

All claims expressed in this article are solely those of the authors and do not necessarily represent those of their affiliated organizations, or those of the publisher, the editors and the reviewers. Any product that may be evaluated in this article, or claim that may be made by its manufacturer, is not guaranteed or endorsed by the publisher.
