# Supplementary material for: Characterization of GmMATE13 in its contribution of citrate efflux and aluminum resistance in soybeans
Source: Front Plant Sci. 2022 Oct 21;13:1027560. doi: 10.3389/fpls.2022.1027560 (PMC9634752; doi:10.3389/fpls.2022.1027560)
Supplement: Supplementary file 1 [file DataSheet_1.docx]

Supplementary Material

**Table S1. Primers sequence for functional study of *GmMATE13*.**

| **Gene** | **Primer (5'-3')** |
| --- | --- |
| *GmMATE13-Luc-F* | CATTCTGGCGGGATCCATGATGCCCCTTTTGATGTT |
| *GmMATE13-Luc-R* | GAGAAAGCTTGGATCCCTAAAGCCCAACATTGTTTACC |
| *GmMATE13-gateway-F* | CAAAAAAGCAGGCTTCATGATGCCCCTTTTGATGTT |
| *GmMATE13-gateway-R* | CAAGAAAGCTGGGTCCTAAAGCCCAACATTGTTTACC |
| *GmMATE13p-GUS-F* | GCAGGCATGCAAGCTTAAAGCGGTTTTTGACTTCTAGCCAC |
| *GmMATE13p-GUS-R* | CTCAGATCTACCATGGCAAGCCCAACATTGTTTACCTTAAGG |
| *GmMATE13-qPCR-F* | TACTGCATTCATTGGCCACATAG |
| *GmMATE13-qPCR-R* | CCTCATTAGAGACCTTCATCAGCAT |
| *GmTublin-F* | GGAAGGCTTTCTTGCATTGGTA |
| *GmTublin-R* | AGTGGCATCCTGGTACTGC |

**Table S2. Physical and chemical properties of four soil types.**

| Sample location | pH | Exchangeable Al content (mol/kg) | Exchangeable Fe content (mol/kg) | Soil types |
| --- | --- | --- | --- | --- |
| Liangcheng, Inner Mongolia | 7.9 | 3.7×10^-11^ | 8.24×10^-8^ | Weak alkaline |
| Changchun, Jilin | 6.4 | 1.3×10^-9^ | 2.37×10^-7^ | Weak acidic |
| Wenzhou, Zhejiang | 5.9 | 3.63×10^-10^ | 1.69×10^-7^ | Acidic |
| Changsha, Hunan | 5.1 | 2.14×10^-7^ | 1.09×10^-7^ | Strong acidic |
